# From productive landscape to cultural space: The heterogeneous coupling of heritage value perception and tourist experience demand in the Grand Canal Cultural Route

**DOI:** 10.1371/journal.pone.0352152

**Published:** 2026-07-29

**Authors:** Di Feng, Shang-chia Chiou, Ying Zhou, Hongyi Li

**Affiliations:** 1 College of Fine Arts, Huaiyin Normal University, Huaian, Jiangsu, China; 2 National Yunlin University of Science and Technology, Douliu, Taiwan; 3 Yancheng Institute of Technology, Yancheng, Jiangsu, China; 4 Nantong Institute of Technology, Nantong, Jiangsu, China; Zhejiang Agriculture and Forestry University: Zhejiang A and F University, CHINA

## Abstract

In the context of rapid urbanization, the transformation of linear hydraulic heritage from productive landscapes to high-quality cultural spaces is a pivotal issue for sustainable development. Taking the Huai’an section of the China Grand Canal as a case study, this research explores the coupling mechanism between heritage value perception and tourist experience demand within a non-Western cultural route. Addressing the insufficient attention paid by existing literature to the dynamic evolution of production-and-transport heritage, this study adopts a systems theory perspective. It utilizes Canonical Correlation Analysis (CCA) to reveal the holistic mapping relationship between two sets of multidimensional variables. The findings indicate that the relationship between heritage value perception and experience demand is not a simple linear correspondence, but rather a significant heterogenetic driving mechanism. Particularly, the strong coupling effect of narrative value and the spirit of place on educational experience demands identifies the core variables through which Grain Transport Cultural Route enhances local identity and spatial perception. The study further reveals the constraints of social stratification on heritage consumption, confirming the moderating effects of cultural capital and social background in the “perception-demand” transformation process. By challenging static preservation views, this study proposes a dynamic, multidimensional evolutionary model of heritage cognition. It provides an analytical framework and policy guidelines for soft-power transformation and segmented protection of linear heritage in non-European contexts, offering a significant theoretical contribution to heritage functional activation in urban sustainable development.

## 1. Introduction

In the era of post-industrial transformation and global urbanization, the conservation of linear cultural heritage is undergoing a fundamental identity reconstruction—shifting from static remains to dynamic cultural assets. However, local heritage development currently faces a profound dilemma: on one hand, excessive commercialization leads to the gentrification-driven loss of heritage landscape genes and the fracturing of community identity [1–3]; on the other hand, the fragmentation of functional remains makes them difficult to embed into modern urban systems, risking resource depletion and the dissolution of historical significance [4,5]. While integrating isolated heritage sites into Cultural Routes has become a consensus for balancing preservation and tourist demand, the existing theoretical framework exhibits a distinct Eurocentric bias [6,7]. The European paradigm, epitomized by the Camino de Santiago, primarily focuses on religious pilgrimages or aesthetically driven linear landscapes [8,9]. Consequently, the theoretical frameworks exhibit significant deficiencies in explaining the dynamic mechanisms behind the transformation of industrial remains with strong productive functions and logistical attributes into modern urban living spaces, such as Grain Transport Cultural Route [10–12]. Particularly for the China Grand Canal, which spans approximately 1,100 kilometers across 10 degrees of latitude, Grain Transport is not merely a static logistical relic but a Flowing Landscape Genealogy. This characteristic presents structural contradictions with contemporary urban expansion [13,14]. Furthermore, existing research predominantly emphasizes heritage value perception [15,16] or canal tourism development models [17,18], yet it often overlooks the deep mechanisms by which the materiality of functional infrastructure translates into urban identity and diversified experience demand through the socio-psychological mapping of tourists. Based on the aforementioned research gaps, this paper aims to fill the conceptual void within the “Global South” context by taking the Huai’an section of the China Grand Canal as an empirical case. The study systematically examines the driving paths from heritage value perception to tourist experience demand. The core Research Question (RQ) is: How do the physical genes of Grain Transport Cultural Route transform into psychological representations through the reconstruction of spatial narratives, thereby triggering the dynamic mechanisms of value perception and differentiated experience demand?

The study perceives the Grand Canal’s Grain Transport system as an urban cultural catalyst, transcending traditional static preservation views. It emphasizes that material genes, such as sluices, dams, and wharves, act as energy sources that—through the construction of cultural routes—trigger a series of socio-psychological chain reactions within urban spaces. Consequently, this process fundamentally reshapes urban cultural identity and the experiential motivations of tourists. The organizational structure of the article is as follows: First, a critical literature review explores spatial justice and gene inheritance in urban governance; second, it outlines the study area and operational definitions of variables; subsequently, Canonical Correlation Analysis (CCA) is employed to empirically evaluate the coupling relationship; finally, the results are discussed to clarify academic controversies regarding productive heritage and to provide theoretical support and policy references for cultural route practices in non-Western contexts.

## 2. Literature review

### 2.1 Paradigm shifts in cultural route theory

The evolution of Cultural Routes theory has witnessed a profound shift in the heritage preservation paradigm, moving from static material islands toward continuous, dynamic systems. Influenced by the Camino de Santiago advocated by the Council of Europe, early scholarship was predominantly confined to the physical continuity of paths and the static preservation of individual nodes [19–21]. However, such a paradigm overlooks the essence of routes as an inherently fluid historical process. In 2008, ICOMOS formally established the status of cultural routes as living landscapes through the Charter on Cultural Routes [22]. Then scholars proposed that cultural routes function as multidimensional spatial networks, the core of which lies in the stratified representation of long-term interactions between humans and their environment [23,24]. In contrast to pilgrimage routes dominated by spiritual meanings, productive-functional routes represented by the Grain Transport demonstrate more significant dynamic characteristics [25–27]. Furthermore, the conservation logic of cultural routes should achieve a transition from static points to dynamic flows, emphasizing the deep symbiosis of material, energy, and cultural flows [28]. Nevertheless, within the context of radical urbanization, these functional paths face severe risks of value fragmentation. Traditional Eurocentric aesthetic frameworks have revealed limitations when interpreting linear heritage characterized by intense production conflicts [29–31]. Another study indicate that interpreting how these material features are transformed into perceived values for modern tourists necessitates the introduction of deeper socio-psychological mechanisms [32]. Therefore, the introduction of a Global South perspective emphasizes the potential of heritage as an inclusive tool for participating in urban renewal [33,34]. This dynamic evolution of paradigmatic dimensions not only reconstructs the theoretical interpretation of the original attributes of Grain Transport Cultural Route but also establishes a logical starting point for analyzing its spatial reconstruction and governance conflicts within contemporary urban systems.

### 2.2 Spatial reconstruction of canal cultural heritage within urban systems

In the context of post-industrial urban transformation, the canal heritage that originally served as a production facility undergoing a significant identity reconstruction toward urban cultural dissemination. Sorenson [35] indicates that remains of the Grain Transport system possess a powerful spatial spillover effect. Through adaptive reuse, these canal relics are not only transformed into urban cultural assets but also serve as local hallmarks that enhance the canal’s overall social value [36,37]. This shift evolves former industrial production paths into contemporary cultural infrastructure. However, as urban spaces expand, a profound tension has emerged between the preservation of landscape genes within canal heritage and modern spatial logic. According to landscape urbanism theory, while heritage is central to urban identity recognition, it frequently faces fragmentation conflicts during contemporary renewal processes [38,39]. Furthermore, the development of canal heritage involves a complex play for spatial rights among diverse stakeholders. From the perspective of spatial justice, imbalanced resource allocation often leads to gentrification, thereby undermining the perceived authenticity of the heritage [40,41]. Consequently, researchers are increasingly calling for a transition from traditional administrative management to collaborative governance, aiming to achieve a harmonious symbiosis among the government, residents, and tourists within the horizon of spatial justice [42]. The evolution of the canal heritage’s external environment, jointly defined by physical spatial reconstruction and social rights dynamics, not only establishes the objective conditions for Grain Transport Cultural Route but also profoundly constructs the cognitive modes and subjective expectations of stakeholders regarding its value.

### 2.3 Drivers and mediation of value perception on tourist experience demand

In the field of heritage tourism, value perception has evolved from a singular evaluative metric into a complex psychological measurement. Throsby [43] provided the foundation for operationalizing the value of heritage in the theory of cultural economy. In the context of canals, heritage value encompasses multiple dimensions including history, aesthetics, society, and economy [44,45]. Crucially, tourist evaluations are not fragmented perceptions, but rather a holistic cognition based on the dual attributes of symbol-function [46,47]. To further explore the underlying mechanism, it is necessary to introduce the theory of Place Attachment. Research has confirmed that when individuals perceive significant value in a heritage site, they develop a strong emotional bond, which in turn drives demand for in-depth experiences [48,49]. As suggested by the theory of expected satisfaction, high levels of value perception heighten the urgency tourists feel for cultural immersion and emotional resonance [50–52]. Nevertheless, perceived authenticity and cultural identity play pivotal mediating roles within this logical chain [53,54]. A tourist’s judgment regarding the authenticity of a heritage site’s living attributes significantly influences the effectiveness of their value evaluation; only when a deep sense of cultural identity is generated can this cognition sublimate into a motivation to pursue cultural authenticity [55–57]. In short, as the primary power source for generating tourist experience demands, value perception intertwines with perceived authenticity and identity to constitute the complex individual behavioral response mechanism in canal heritage tourism.

In conclusion, although current scholarship has established a theoretical foundation for the evolution of cultural routes and heritage perception, significant research gaps remain within the field of Grain Transport Cultural Route. First, a misalignment persists in theoretical paradigms; mainstream research predominantly follows the aesthetic paradigm of European, failing to adequately represent the dynamic evolutionary logic of Chinese Grain Transport Cultural Route as a productive-functional living landscape under conditions of intense urbanization conflict. Secondly, the transmission mechanism is still unclear. The psychological process by which the spatial characteristics of the physical world are transformed into specific experiential needs through local attachment remains to be explored. In particular, there is a lack of systematic empirical evidence regarding the mediating effect of the perception of authenticity and cultural identity in this causal chain. Therefore, this study is based on the “Global South” context and aims to bridge the aforementioned theoretical gaps. By integrating the Cultural Capital Theory with environmental psychology models, this paper aims to empirically investigate the driving pathways of Grain Tansport heritage value perception on tourist experience demand, while validating the moderating effects of identity and authenticity. This not only helps to deepen the theoretical interpretation of the dynamic evolution of linear cultural landscapes but also provide scientific decision-making support for the collaborative governance and sustainable utilization of large-scale functional heritage.

## 3. Materials and methods

### 3.1 Study area

**Fig 1:**
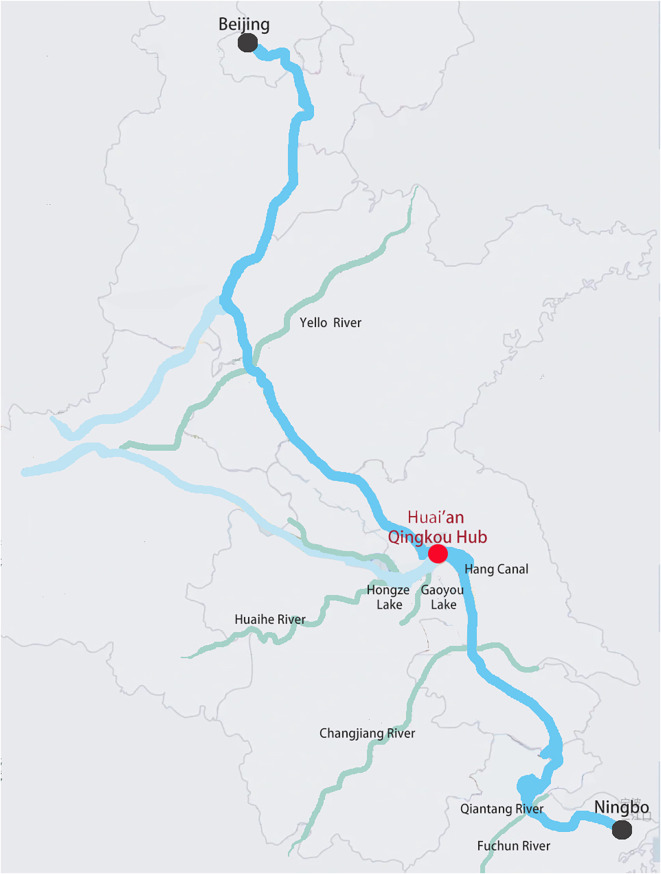
Huai ‘an Qingkou Hub and the Grand Canal (Source: Created by the author).

**Fig 2:**
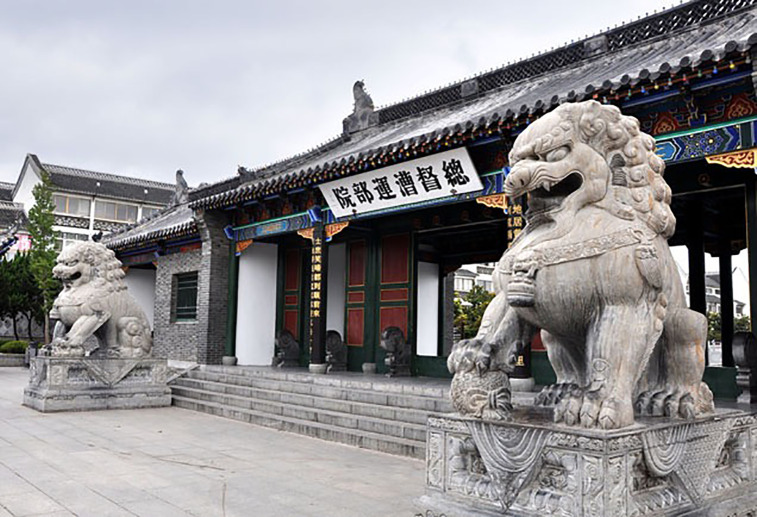
The Governor’s Office of Huai ‘an Grain Transportation (Source: http://www.zghaq.gov.cn/).

The selection of the Huai’an section of the Grand Canal as the primary research site is justified by its unique position as a technological, spatial, and psychological nexus (Fig 1). Historically, as the confluence of the Yellow, Huai, and Grand Canal, Huai’an served as the seat of the Governor-General of Grain Transport—the supreme administrative body of the system—making it a concentrated spatio-temporal repository of productive landscape genes [58–60] (Fig 2). From a spatial and structural perspective, this region epitomizes the tensions of radical urbanization. The intense pressure of urban sprawl has fragmented originally continuous transport paths into isolated nodes, creating a representative contestation field. This environment offers a unique opportunity to explore how heritage, acting as an urban catalyst, can re-anchor spatial justice and transition from static preservation to the activation of dynamic flow-landscapes [40,61,62].

More importantly, Huai’an provides an ideal laboratory to examine the materiality–perception–demand transformation mechanism. In the post-industrial context, the region’s heritage is undergoing a profound identity reconstruction—from peripheral engineering facilities to core urban cultural assets [63,64]. Through the mechanism of place attachment, these physical remains (e.g., sluices and dams) are elevated from historical relics to symbols of cultural authenticity in the psychological representation of visitors [65]. By analyzing this section, the study can precisely decode how functional landscape genes are converted into perceived value through the mediation of authenticity and cultural identity [66].

In summary, the Huai’an section’s dynamic expansionary character aligns perfectly with the core mission of investigating the nexus between heritage value perception and experiential demand, offering an irreplaceable empirical reference for the collaborative governance of linear heritage in non-Western contexts.

### 3.2 Variables and data

This study employs Canonical Correlation Analysis (CCA) to investigate the structural correlations between heritage value perception and tourist experience demand along the cultural route on Huai’an Grain Transport. The decision to utilize CCA over simple regression analysis stems from its holistic perspective, which allows for capturing the overall mapping relationship between two multidimensional variable sets. This approach is superior to single-path analysis in reflecting the complexity of cultural routes as linear, dynamic landscape systems [67,68]. To ensure logical rigor and eliminate the risk of circular reasoning, variables were operationally reconstructed based on the ICOMOS Charter on Cultural Routes and relevant heritage perception theories [69,70]. The Heritage Value Perception (independent variable set) includes: Narrative Value: Measuring the logical connectivity of the route regarding historical Grain Transport events; Place Value: Referring to the cultural connotations and atmosphere carried by the heritage spatial environment; Tourism Value: Assessing the potential of resources to be transformed into high-quality attractions; Economic Value: Perceived contribution of heritage to local industries and employment; Historical Value: Focusing on the cognition of the heritage’s objective function in verifying history. The Tourist Experience Demand (dependent variable set) is categorized based on Experience Economy theory [71] into: Aesthetic Experience: Appreciation of linear landscape aesthetics; Leisure Experience: The desire for psychological relaxation and interaction within cultural spaces; Educational Experience: The aspiration to acquire local knowledge and understand the pulse of civilization; Service Experience: Functional requirements for the completeness of supporting facilities.

**Fig 3:**
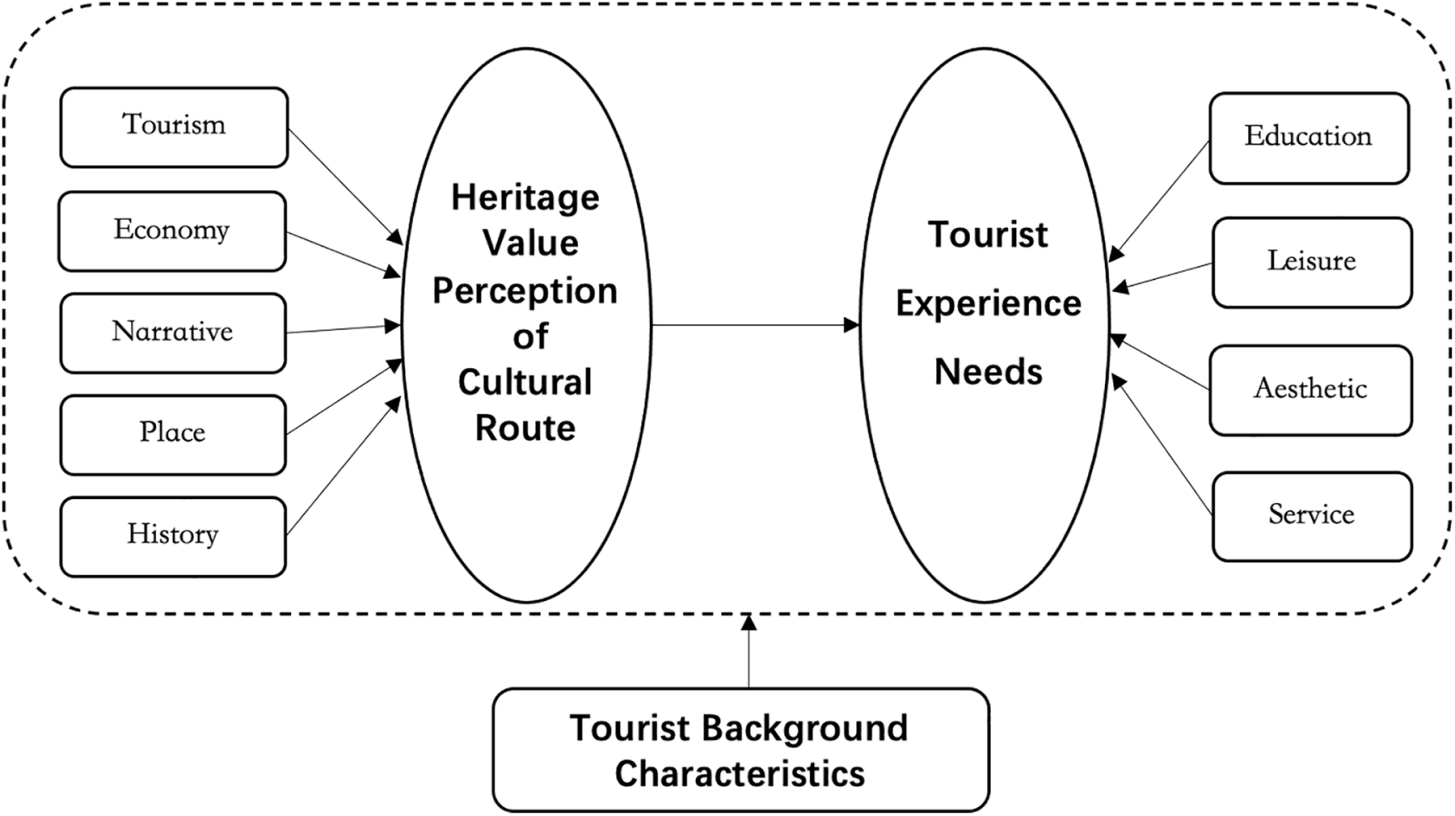
Research hypothesis (Source: Created by the author).

Based on the heterogeneous characteristics of cultural routes and a systems theory perspective, this study moves beyond simple demographic descriptive hypotheses toward exploring the deep logic between variable sets, proposing the following hypotheses:

H1: There is a significant canonical correlation between the heritage value perception set and the experience demand set of the Grain Transport Cultural Route, indicating a systematic and holistic mapping relationship.
H2: Different dimensions of value perception trigger demand dimensions following a significant heterogeneity pattern, where specific perception dimensions (e.g., narrative and historical values) differentially drive specific psychological or functional demands (e.g., educational and aesthetic experience).
H3: The social background of tourists (e.g., residential distance, cultural familiarity) significantly moderates the strength and direction of the perception-demand mapping pattern through social stratification effects (Fig 3).

To validate these hypotheses, the study extracts optimal linear combinations via Principal Component Analysis (PCA) and utilizes a pilot test to eliminate overlapping indicators that could confuse causal logic, ensuring that independent variables (objective cognition) and dependent variables (subjective demand) remain logically independent. To address the limitations of cross-sectional data in causal inference, demographic characteristics are introduced as control variables, and collinearity tests are employed to mitigate the risk of spurious correlations. This approach allows for a profound analysis of the associative patterns through which heritage values of Grain Transport Cultural Route are transformed into specific experience demands among diverse tourist groups.

## 4. Research results

### 4.1 Descriptive statistics of demographic variables for the formal questionnaire

After conducting the statistical analysis of the pre-test, the quality and reliability of the questionnaire were confirmed. The final questionnaire was distributed from February 22, 2025, to March 6, 2025. The questionnaire begins with a written consent request from the participants, ensuring that all participants are fully informed and participate voluntarily.Tourists’ backgrounds, including educational level, occupation, marital status, and income, influence their travel intentions [72]. Furthermore, the questionnaire screened participants based on their frequency of travel and familiarity with Grain Transport to further understand the impact of these factors on the perception of canal cultural values. Ultimately, a total of 1,292 questionnaires were collected; after excluding invalid samples, 1,066 valid samples remained, resulting in an effective recovery rate of 82.5%. Based on the valid questionnaire data, statistical analysis was conducted, and the distribution of various demographic variables is presented in Table 1.

**Table 1:** Distribution of demographic variables.

Item	Division	Number	Percentage
Gender	Male	445	41.7%
	Female	621	58.3%
Age (years)	18-25	303	28.4%
	26-39	424	39.8%
	40-59	276	25.9%
	60+	63	5.9%
Educational Level	Less than high school	55	5.2%
	High school diploma	107	10.0%
	College degree or Graduate degree	673	63.1%
	Master degree or above	231	21.7%
Occupation	Student	249	23.4%
	Public institutions	315	29.5%
	Government agencies	74	6.9%
	Business unit	231	21.7%
	Entrepreneur	55	5.2%
	Other	142	13.3%
Marital Status	Unmarried	457	42.9%
	Married	584	54.8%
	Divorced	16	1.5%
	Widowed	9	0.8%
Monthly Salary	Less than 3000 yuan	339	31.8%
	3001-5000 yuan	244	22.9%
	5001-8000 yuan	227	21.3%
	8001-12000 yuan	153	14.4%
	12001-18000 yuan	50	4.7%
	Over 18001 yuan	53	5.0%
Number of Trips	0 times	179	16.8%
	1-3 times	777	72.9%
	More than 4 times	110	10.3%
Travel Area	Domestic tourism	1007	94.5%
	International travel	59	5.5%
Familiarity with the Grain Transport Culture	Have no idea	250	23.5%
	heard of	413	38.7%
	Have a little understanding	293	27.5%
	Familiar	77	7.2%
	Very familiar	33	3.1%

### 4.2 Heritage value perception

Based on the collected data, the importance of the five factors within the heritage value dimension was ranked as follows: Historical (4.31), Tourism (4.27), Narrative (4.26), Economic (4.26), and Place (4.23). Reliability analysis was conducted on these factors, yielding Cronbach’sαvalues all greater than 0.8, which indicates that the reliability of cultural route value perception meets the required academic standards. Consequently, the heritage value perception of cultural routes can be validly discussed across these five dimensions.

### 4.3 Tourist experience demand

The data analysis revealed that the importance ranking of the four factors for tourist experience demands is: Service (4.37), Educational (4.34), Aesthetic (4.28), and Leisure (4.22). Reliability analysis showed that the Cronbach’sαvalues for these factors all exceeded 0.8, confirming that the reliability of tourist experience demands in the questionnaire is consistent with standards. Therefore, the tourist experience demands of cultural routes can be effectively analyzed through these four dimensions.

### 4.4 Average variance explained and canonical correlation

As shown in Table 2, the first canonical variable for the independent set (λ1) explains 75.4% of the variance across the five variables: Historical, Narrative, Tourism, Economic, and Place values. Meanwhile, the first canonical variable for the dependent set (η1) explains 78.0% of the variance across the four variables: Educational, Leisure, Aesthetic, and Service. Regarding statistical significance, the first pair of canonical correlation coefficients reached the highest value of 0.828, with a significance level of p = 0.000 < 0.05, and an explanatory power of 68.6%. Generally, a mutual explanatory power exceeding 5% is considered viable for retention. In this study, the independent variable set (heritage value perception of cultural routes), through the first pair of canonical variables (λ1 and η1), can explain 53.4% of the total variance in the dependent variable set (tourist experience demands).

**Table 2:** Canonical correlation analysis between cultural route value and tourist experience needs.

Variable λ	Canonical Variables			Variable η	Canonical Variables		
	λ1	λ2	λ3		η1	η2	η3
Historical	0.365	−1.310	−0.642	Education	0.482	0.758	−0.704
Narrative	0.224	1.558	0.028	Leisure	0.199	0.096	1.415
Tourism	0.281	0.685	−0.144	Aesthetic	0.194	0.627	−0.342
Economy	0.129	−0.453	−0.669	Service	0.254	−1.531	−0.212
Place	0.116	−0.489	1.555				
The Percentage of Variance	75.4%	7.7%	9.9%	The Percentage of Variance	78.0%	4.4%	6.5%
Overlap	51.7%	0.2%	0.1%	Overlap	53.4%	0.1%	0.1%
**ρ** ^**2**^					0.686	0.020	0.009
Canonical correlation					0.828	0.140	0.093
Sig					0.000 *	0.000 *	0.023 *

* P < 0.005.

### 4.5 Canonical correlation in regression analysis

**Fig 4:**
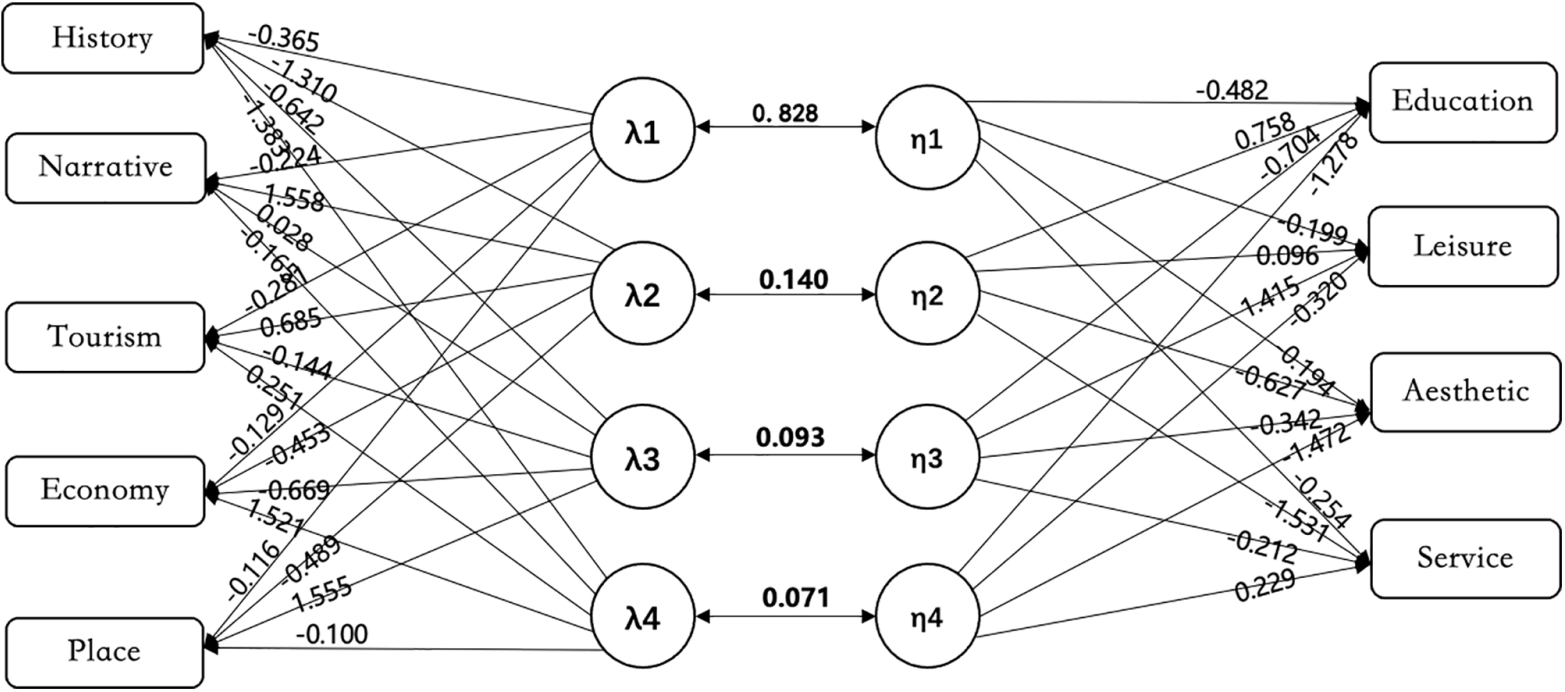
Canonical Correlation Analysis of cultural route value and tourist experience demand (Source: Created by the author).

Through Canonical Correlation Analysis (CCA), this study constructed a multiple mapping model between the heritage value perception set and the tourist experience demand set for Grain Transport Cultural Route (Fig 4). The results reveal four pairs of significant canonical correlation channels between the two groups of variables. Notably, the first canonical correlation coefficient reaches as high as 0.828, providing robust evidence for the significant holistic mapping effect of heritage value perception on experience demands (H2). Observation of the heterogeneity patterns through path loadings indicates that Historical Value and Place Value contribute significantly to the first canonical variable (λ1). These factors map precisely onto the demand dimension dominated by Educational Demand (η1), confirming that the profound perception of Grain Transport historical context and spatial atmosphere serves as the core driver for tourists’ knowledge-seeking needs (H3). Meanwhile, the complex distribution of path weights within the model reveals a non-equilibrium triggering mechanism across different value dimensions—such as aesthetic, leisure, and service demands—reflecting the structural interconnectedness within the landscape system. This many-to-many networked mapping structure not only avoids the partiality of single-causality explanations but also uncovers, from a holistic perspective, how the values of Grain Transport Cultural Route are heterogeneously transformed into a multidimensional system of tourist experience drivers.

According to the results of the canonical analysis, the study primarily references the linear relationship between the first pair of canonical variables. The specific influence relationship is expressed as follows:


$$\begin{array}{c} \lambda \text{1}=\eta \text{1}, \end{array}$$

where


$$\begin{array}{c} \lambda \text{1}=\text{0}.\text{365}*\text{Historical}+\text{0}.\text{224}*\text{Narrative}+\text{0}.\text{281}*\text{Tourism}+\text{0}.\text{129}*\text{Economy}+\text{0}.\text{116}*\text{Place}; \end{array}$$

$$\begin{array}{c} \eta \text{1}=\text{0}.\text{482}*\text{Education}+\text{0}.\text{199}*\text{Leisure}+\text{0}.\text{194}*\text{Aesthetics}+\text{0}.\text{254}*\text{Service}. \end{array}$$

### 4.6 Interaction effects of background variables and social stratification analysis

The study further explores the profound impact of tourist background characteristics on heritage value perception (Table 3) and experience demands (Table 4) through Analysis of Variance (ANOVA). Departing from previous single-dimensional descriptive studies, this research emphasizes the analysis of interaction effects among multiple variables to uncover the heterogeneous characteristics of tourist demands within the context of social stratification [73].

**Table 3:** ANOVA test results for background information and heritage value perception.

Source	Type III SS	df	MS	F	Sig.	Partial Eta Squared
Modified Model	19.370a	28	0.692	2.901	0	0.073
Intercept	2417.934	1	2417.934	10139.463	0	0.907
Age	3.308	3	1.103	4.624	0.003*	0.013
Educational Level	0.578	3	0.193	0.796	0.496	0.002
Monthly Salary	3.072	5	0.614	2.548	0.027*	0.013
Familiarity with the Grain Transport Culture	6.302	4	1.576	6.626	0.000*	0.028
Age*Educational Level	4.154	9	0.462	1.936	0.044*	0.017
Marital status*Monthly Salary	6.522	11	0.593	2.459	0.005*	0.027
Gender*Age*Educational Level	4.751	6	0.792	3.32	0.003*	0.019
Occupation*Marital Status*Monthly Salary	8.402	21	0.4	1.659	0.031*	0.034

* P < 0.005.

**Table 4:** ANOVA test results for background information and tourist experience needs.

Source	Type III SS	df	MS	F	Sig.	Partial Eta Squared
Modified Model	18.029a	28	0.644	2.867	0	0.072
Intercept	2441.177	1	2441.177	10871.425	0	0.913
Age	1.929	3	0.643	2.863	0.036*	0.008
Occupation	3.09	5	0.618	2.794	0.017*	0.033
Educational Level	4.632	3	1.544	6.658	0.000*	0.028
Familiarity with the Grain Transport Culture	4.67	4	1.167	5.193	0.000*	0.021
Age*Educational Level	5.569	9	0.619	2.755	0.003*	0.023
Occupation*Educational Level	2.697	7	0.385	2.395	0.035*	0.259
Educational Level*Monthly Salary	6.036	14	0.431	1.918	0.021*	0.027
Marital Status*Monthly Salary	4.705	11	0.428	1.867	0.040*	0.02
Number of Trips*Familiarity with the Grain Transport	5.835	12	0.486	2.118	0.014*	0.024

* P < 0.005.

#### 4.6.1 Multiple social constraints on value perception.

The results in Table 3 indicate that age (Sig. = 0.003), economic income (Sig. = 0.027), and familiarity with Grain Transport culture (Sig. = 0.000) all exert significant main effects on value perception. A more profound finding, however, is the significant three-way interaction effect of “Gende*Age*Education level” (Sig. = 0.003).This reflects a complex socio-psychological mechanism: middle-aged and elderly female tourists with higher education backgrounds exhibit the highest levels of perception regarding the historical value of Grain Transport Cultural Route. This phenomenon can be interpreted through the lenses of Cultural Capital and social memory—this specific group tends to possess stronger historical empathy and is more inclined to view the Grain Transport Cultural Route as an informal educational space that integrates both aesthetic appreciation and intellectual verification.

#### 4.6.2 Heterogeneous triggering patterns of experience demands.

The data in Table 4 further confirm the complexity of social backgrounds in driving experience demands. Beyond single factors such as occupation and familiarity, the interaction effects of “Number of Trips * Familiarity with Grain Transport” (Sig. = 0.014) and “Education Level * Monthly Salary” (Sig. = 0.021) significantly influence tourists’ expressions of demand. Notably, the interaction of “Occupation $\times$ Education Level” exhibits exceptionally high explanatory power. This provides robust support for the study’s argument regarding social stratification: for professional technicians or intellectual groups with high educational backgrounds, experience demands are no longer limited to basic leisure or aesthetics. Instead, they display strong heterogeneous demand characteristics—specifically, an intense craving for heritage authenticity and deep narrative.

#### 4.6.3 Hypothesis validation.

The aforementioned findings provide empirical validation for research hypothesis H3. The results of the ANOVA extend beyond the simple influence of background variables on perception, revealing how combinations of different social attributes reshape the cognitive maps of tourists. This stratified variance implies that the development of Grain Transport Cultural Route should not follow a uniform model. Instead, it must precisely align with specific demand profiles woven from educational backgrounds, economic capacities, and social identities. By treating background variables as control variables and conducting a deep analysis of their interaction effects, this study has successfully isolated the interference of demographic characteristics. Consequently, it has more accurately identified how different types of value perception heterogeneously trigger experience demands within specific social groups.

## 5. Discussion

This study explores the structural associations between the heritage value perception of the Huai’an Grain Transport Cultural Route and tourist experience demands. The results indicate that the relationship between the two is not a simple linear mapping but rather a dynamic system co-constructed through complex psychological and cultural mechanisms within a context of social stratification.

### 5.1 Mechanism analysis of core associations: The logical transformation from cognition to demand

Canonical Correlation Analysis (CCA) confirms the significant holistic driving effect of heritage value perception on experience demands (H2). Specifically, the strong mapping relationship between narrative value and place value toward educational experience demands reveals the core mediating roles of perceived authenticity and sense of place. Unlike traditional mass tourism, the Grain Transport Cultural Route as a form of fluid heritage that derives its core appeal not from isolated physical landscapes, but from the continuity of its historical logic. When tourists perceive a strong historical narrative and spatial atmosphere, it triggers a desire for trans-temporal dialogue, thereby transforming simple sightseeing behavior into an active demand for deep learning. This transformation mechanism suggests that the awakening of heritage conservation awareness is not an isolated process; rather, it is converted into a pursuit of high-quality heritage products by enhancing the cultural involvement of tourists.

### 5.2 Interaction effects under social stratification: Sociological roots of heterogeneous demands

The multiple interaction effects identified in the ANOVA profoundly reveal the constraints of social stratification on heritage consumption. The finding that highly educated middle-aged and elderly women exhibit the strongest perception of historical value reflects this group’s unique social capital and role positioning. From a sociological perspective, this demographic often plays the role of cultural transmitter within the family, tending to view Grain Transport Cultural Route as a second classroom for the education of children or grandchildren. Their high sensitivity to historical value is, in essence, a subconscious expression of social class continuity and cultural capital accumulation. By contrast, the divergence between professional technical groups and general tourists regarding aesthetic authenticity versus commercial service demands reveals a deep-seated stakeholder conflict in heritage utilization: namely, the tension between the elite class’s aesthetic pursuit of pure cultural preservation and the general public’s demand for tourism convenience.

### 5.3 Critical reflection: The authenticity crisis and comparative cross-case analysis

The insufficient mapping intensity of certain sub-dimensions in the model reflects the authenticity challenges currently facing the development of the Huai’an Grain Transport Cultural Route. Although overly modernized urban landscape development (such as homogenized pseudo-classic commercial streets) has boosted the local economy, but to some extent, it has weakened the historical sense of vicissitude inherent in the heritage. This leads to a disconnect where tourists find it difficult to equate economic benefits with aesthetic value. In comparison with international cases such as Colombo or the Nile, the Huai’an case exhibits distinct characteristics of top-down administrative promotion. While this model can rapidly enhance visibility, it remains deficient in stimulating emotional resonance among grassroots tourists. This suggests that the focus of cultural route construction urgently needs to shift from the mere accumulation of landscapes to the creation of emotional linkages.

## 6. Conclusion

Through Canonical Correlation Analysis, this study refines an Evolutionary Framework of Cultural Route Cognition. This model posits that the experiential logic of cultural routes follows a systematic evolution: moving from objective narrative cognition to spatial-place resonance, and finally to the transformation of heterogeneous demands. As linear landscapes, the holism of Grain Transport Cultural Route takes precedence over their locality. This finding provides new theoretical support for understanding the functional reactivation of linear cultural heritage within modern urban spaces. Based on the value-demand heterogeneity triggering patterns identified in this research, the following prioritized management recommendations are proposed:

(1) Prioritize digital narrative restoration over indiscriminate infrastructure construction: Given that narrative value significantly drives educational demand, resources should be prioritized for the digital scene reconstruction of hydraulic heritage—such as sluices, dams, and river cross-sections. Utilizing AR/VR technology to recreate the grandeur of historical Grain Transport is more effective than large-scale modern commercial development.
(2) Enhance visual visibility to activate community cultural identity: Priority should be given to restoring physical nodes with strong Place Value. By improving spatial identifiability, management can activate place attachment among surrounding residents and tourists, thereby achieving long-term social benefits for heritage conservation.
(3) Implement precise, segmented promotion strategies based on social stratification: For groups with high cultural capital, heritage co-creation programs should be established to transform their social circles into informal cultural classrooms. For younger demographics, dry historical facts should be converted into highly interactive leisure products to bridge the gap between high cognitive awareness and low participation levels.

### 6.1 Limitations and future research prospects

While this study reveals correlations between the two sets of variables, certain limitations remain. First, constrained by cross-sectional data, this study faces challenges in establishing strict causal inference. Future research should introduce Structural Equation Modeling (SEM) to empirically test the mediating effects of place identity or perceived authenticity in the cognitive transformation process. Second, this study does not cover the long-term evolution of cultural routes during rapid urban expansion. Research should adopt a Longitudinal Study design, utilizing time-series data to explore the dynamic flux of tourist perceptions across different policy cycles, thereby providing more predictive decision support for the sustainable development of the Grand Canal Cultural Belt.

## Supporting information

S1 Data Supporting information-dataset. (XLSX)
